# Discriminative Power of EuroSCORE in Predicting Morbidity and Prolonged Hospital Stay in an Iranian Sample Population

**Published:** 2014-01-12

**Authors:** Mahdi Najafi, Mehrdad Sheikhvatan, Mahmood Sheikhfathollahi

**Affiliations:** *Tehran Heart Center, Tehran University of Medical Sciences, Tehran, Iran.*

**Keywords:** *Coronary artery bypass* • *Risk assessment* • *ROC curve*

## Abstract

***Background: ***The EuroSCORE is a simple and rigorous risk stratification model and is, thus, commonly used in predicting the early and late outcomes of cardiac surgery across the world. We aimed to assess the discriminative power of the EuroSCORE model to predict postoperative morbidity and total prolonged length of stay in hospital (LOS) and Intensive Care Unit (ICU) stay in an Iranian group of cardiac surgical population.

***Methods: ***In a prospective study, the additive EuroSCORE model was applied to 570 patients undergoing isolated coronary artery bypass grafting (CABG) at Tehran Heart Center. The discrimination power of the EuroSCORE model was tested by the area under the receiver operating characteristic (ROC) curve and the calibration by comparing the observed and predicted outcomes across the risk spectrum assessed using the Hosmer-Lemeshow goodness-of-fit test.

***Results: ***The mean age was 59.03 ± 0.73 years and 429 out of the 570 (75.3%) patients were men. The overall morbidity rate was 47.5%. The observed morbidity in the high-risk patients (EuroSCORE > 6) was significantly greater than that in the low-risk patients (EuroSCORE ≤ 6). Furthermore, 51.2% of the patients had LOS beyond 14 days. Both prolonged LOS (> 14 days) and prolonged ICU stay (> 72 hours) were more prevalent in the high-risk group than in the low-risk group. The discriminative power of the EuroSCORE in predicting morbidity, prolonged LOS, and ICU stay was poor with an area under the ROC curve of 0.617, 0.598, and 0.581, respectively. However, this risk score showed good calibrations for morbidity (p value = 0.119), prolonged LOS (p value = 0.958), and prolonged ICU stay (p value = 0.620).

***Conclusion: ***The EuroSCORE provided inappropriate discrimination in predicting early morbidity and prolonged LOS and ICU stay in our study population. Creating a revised model may enable us to accurately predict outcomes in Iranian CABG patients.

## Introduction

Detection of the changes and differences in risk profiles could effectively lead to the best postoperative outcome and as such prevent inappropriate long-term events and optimize the use of limited healthcare resources.^1^^-^^3^ One of the most simple and rigorous risk stratification models is the European system for cardiac operative risk evaluation score (EuroSCORE). The EuroSCORE was proposed primarily for risk estimation in coronary artery bypass grafting (CABG).^4^

This risk management tool was constructed from the data analysis of 19030 patients from 128 centers across the whole of Europe and is organized as two additive and logistic models. In the logistic model, 17 risk variables and a beta coefficient associated with each variable are used to provide the likelihood of death for any patient. A simpler variant of the logistic model is also available as the additive EuroSCORE, which assigns a weight to each risk factor presented by the patient and the sum of the weights provides the likelihood of dying for the patient.^5^^, ^^6^

The EuroSCORE has been validated for its ability to predict early and long-term mortality, major complications, and prolonged in-hospital length of stay (LOS) both in the whole context of cardiac surgery and in isolated CABG.^7^^-^^10^ The EuroSCORE has also been previously shown to be reliable in assessing costs and patients’ quality of life after CABG.^11^^, ^^12^

Any risk scoring system may only be used reliably when its validity and performance have been tested in the local patient population.^13^ Although the EuroSCORE is perhaps the most common tool used for risk stratification in patients undergoing cardiac surgeries in our country, it has not been validated enough in the Iranian population. Therefore, recruiting an Iranian group of cardiac surgical patients, we assessed the discriminative power of the EuroSCORE model to predict postoperative morbidity and total prolonged LOS and Intensive Care Unit (ICU) stay.

## Methods

We performed a prospective study on a total of 570 consecutive patients undergoing CABG at Tehran Heart Center from May 2006 up to five month later. The data set was restricted to first-time isolated CABG subjects. Those who underwent CABG combined with a heart valve repair or replacement, resection of a ventricular aneurysm, or other surgical procedures were excluded. The study was approved by the local institutional Ethics Committee, and written informed consent was obtained from all the patients.

To retrieve those variables included in the EuroSCORE risk scoring method, a specific questionnaire was filled out for each patient to collect data by interviewing at the first visit on the admission day. Patient-related factors, preoperative clinical state, and cardiac-related factors were recorded according to the EuroSCORE criteria. The additive version of the EuroSCORE was employed to predict morbidity and prolonged LOS in the studied patients. In this version, the probability of death for every patient was calculated by summing the relative weights for each risk factor. Based on this estimation, there were 506 patients in the low-risk group (additive EuroSCORE risk ≤ 6) and 64 patients in the high-risk group (additive EuroSCORE risk > 6).

In the present study, in-hospital postoperative morbidity was defined as the existence of at least one of these complications: wound infection, postoperative arrhythmias, myocardial infarction (MI), respiratory failure, or brain stroke. Prolonged LOS was also defined as a total LOS of > 14 days and prolonged ICU stay of > 72 hours. We did not consider the validation of the EuroSCORE to predict mortality because only 3 deaths (mortality = 0.5%) occurred within 30 days following surgery.

The continuous data are shown as means and standard deviations, and the categorical variables are presented as percentages. The validity of the additive regression model was examined using the Hosmer-Lemeshow goodness-of-fit test. The test derives a χ^2^ statistic from the differences between the observed and expected values for morbidity and prolonged LOS across different risk spectrum. An acceptable calibrated model yields a low χ^2^ and a corresponding p value > 0.05, indicating acceptable calibration of the model. Accordingly, it accurately predicts the two outcomes mentioned above.^14^

The model’s ability to discriminate between possible outcomes was assessed in terms of its capacity to distinguish between patients with and without morbidity or prolonged LOS during hospitalization. Discriminatory capacity was analyzed using the calculation of the c-index and is presented with a 95% confidence interval. 

The c-index reflects the ability of the model to discriminate between possible outcomes (e.g. dead vs. surviving cases). Discrimination refers to the ability of a model to distinguish value 0 from value 1 of the dependent variable. In other words, it is the ability of the score to distinguish patients who died from those who lived. Discrimination can be assessed by the area under the receiver operating characteristic (ROC) curve. The ROC area can be interpreted as the probability that a patient who died had a higher risk score than a patient who survived. Thus, the area under the curve is the percentage of randomly drawn pairs for which this is true. This is a fairly subjective measure and values > 0.8 usually indicate potentially useful discrimination. A value of 0.5 indicates random predictions. A useless model would have a c-index of 0.5, indicating that the model would predict one outcome to be just as likely as any other. A c-index of 1.0 would be found in a "perfect" ideal model. Generally, the discriminative power of the model is thought excellent if the area under the ROC curve is > 0.80, very good if > 0.75, and good if > 0.70.^15^

Validation analysis was carried out using the STATA statistical package (version 8.0; College Station, TX, USA) and comparative analysis using SPSS (version 13.0, SPSS Inc., Chicago, IL, USA). All p-values were two-sided, with statistical significance defined by a p value ≤ 0.05.

## Results

Comparing the risk factors between the EuroSCORE patients and our patients (Table 1), the distribution of the female gender was similar in both populations; however, our patients were younger. In our study participants, the history of obstructive pulmonary disease, neurological dysfunction, unstable angina, and recent myocardial infarction was more prevalent than that in the EuroSCORE patients.

Among the patients undergoing cardiac surgery within the study period, an overall morbidity rate of 47.5% was observed and the rate of morbidity was significantly greater in the high-risk patients than in the low-risk subjects (Table 2). Furthermore, over half of the patients had LOS > 14 days, whereas ICU stays > 72 hours occurred in 17%. Both prolonged LOS and prolonged ICU stay were more common in the high-risk group compared to the low-risk group (Table 2).

The discriminatory power of the EuroSCORE using the area under the ROC curve was 0.617 (95%CI; 0.571 – 0.663) for morbidity, 0.598 (95%CI; 0.552 – 0.645) for LOS >14 days, and 0.581 (95%CI; 0.518 – 0.644) for ICU stay > 72 hours, which was deemed quite low. However, the additive EuroSCORE indicated good calibrations for morbidity (Hosmer–Lemeshow; χ^2^ = 8.761; p value = 0.119), LOS > 14 days (Hosmer–Lemeshow; χ^2^ = 1.062; p value = 0.958), and ICU stay > 72 hours (Hosmer–Lemeshow; χ^2^ = 3.520; p value = 0.620).

**Table 1:** Risk factors prevalence in the EuroSCORE original database compared to Iranian CABG patients in our study

Risk Factors	EuroSCORE Population (%) (n = 19,030)	Iranian CABG Population (%) (n = 570)	P value
Age (y)			
< 60	33.2	51.2	< 0.001
60-64	17.8	19.8	0.219
65-69	20.7	16.0	0.006
70-74	17.9	9.5	< 0.001
> 74	9.6	3.5	< 0.001
Female gender	27.8	24.7	0.103
Creatinine > 200 µmol/L (> 2.26 mg/dL)	1.8	1.1	0.213
Extra-cardiac arteriopathy	11.3	27.7	< 0.001
COPD	3.9	6.7	< 0.001
Neurological dysfunction	1.4	3.9	< 0.001
Previous cardiac surgery	7.3	0.9	< 0.001
Active endocarditis	1.1	0	0.012
Critical preoperative state	4.1	5.4	0.125
Unstable angina	8.0	40.1	< 0.001
Ejection fraction (%)			< 0.001
30-50	25.6	60.4	< 0.001
< 30	5.8	2.3	< 0.001
Recent MI	9.7	35.9	< 0.001
Pulmonary hypertension	2.0	0	< 0.001
Emergency surgery	4.9	13.5	< 0.001
Ventricular septal rupture	0.2	0	0.285
Thoracic aortic surgery	2.4	0	< 0.001

CABG, Coronary artery bypass grafting; COPD, Chronic obstructive pulmonary disease; MI, Myocardial infarction

**Table 2:** Prevalence of morbidity, prolonged total length of stay (LOS) in hospital, and prolonged intensive care unit (ICU) stay in high- and low-risk groups according to EuroSCORE^*^

	Observed event % (95%CI)	P value
Morbidity (n = 271)		0.003
EuroSCORE ≤ 6 (n = 506)	45.3 (41.0 – 49.6)	
EuroSCORE > 6 (n = 64)	65.6 (54.0 – 77.2)	
Total (n = 570)	47.5 (43.4 – 51.6)	
		
Prolonged total LOS^**^ (n = 292)		0.001
EuroSCORE ≤ 6 (n = 506)	48.8 (44.4 – 53.2)	
EuroSCORE > 6 (n = 64)	70.3 (59.1 – 81.5)	
Total (n = 570)	51.2 (47.1 – 55.3)	
		
Prolonged ICU stay^***^ (n = 97)		0.031
EuroSCORE ≤ 6 (n = 506)	15.8 (12.6 – 19.0)	
EuroSCORE > 6 (n = 64)	26.6 (15.8 – 37.4)	
Total (n = 570)	17.0 (13.9 – 20.1)	

* Data are presented as % (95%CI)

** Is defined as a total length of stay of > 14 days

*** Is defined as an ICU stay of > 72 hours

## Discussion

This study suggested that the EuroSCORE provided inappropriate discrimination for early morbidity, prolonged LOS, and ICU stay in our population. The range of predictions provided by the EuroSCORE for the early outcome of CABG in our sample seemed to be limited in part due to the fact that most of our study patients were assigned to the low- risk group (EuroSCORE ≤ 6), and only 11.2% of the patients were classified as higher-risk cases (EuroSCORE > 6). This is reflected in the relatively low ROC area.^1^ Therefore, this study showed that the EuroSCORE provides an irrelevant estimate of the early outcome after CABG in an Iranian population. Our findings are in accordance with the only other study among Iranian patients by Sadeghi et al.^16^ Nonetheless, to the best of our knowledge, this is the first prospective study on the additive EuroSCORE performance among Iranian CABG candidates. 

The results of studies about the EuroSCORE performance are not consistent in the different parts of the world. In a similar study by Nilsson et al.,^11^ the Hosmer-Lemeshow p-value for the EuroSCORE prediction of prolonged ICU stay indicated good accuracy and the area under the ROC curve was also acceptable Noyez et al.^17^ found that the EuroSCORE was not of value as a predictive system for prolonged LOS, but a significant relationship was observed between the high-risk patients identified by the EuroSCORE and prolonged ICU stay. Yap et al.^18^ found that the additive and logistic EuroSCORE models of risk prediction could not accurately predict the outcomes of patients undergoing cardiac surgery in Australia and that the calibrations of both models were poor. Among the Chinese population, the EuroSCORE model did not also accurately predict outcomes after CABG.^19^ A large study in Turkish people revealed that the original EuroSCORE cannot predict mortality accurately.^20^

Problems for the definition and development of a suitable discriminatory system may be because of the wide range of complications registered under morbidity and the possible different impact of these complications on ICU and hospital stays.^17^ In some studies, the EuroSCORE had a good discriminative power and appropriate calibration in predicting postoperative renal failure, sepsis and/or endocarditis, respiratory failure, and prolonged length of stay, but it was unable to predict other major complications such as intraoperative stroke, stroke over 24 hours, postoperative MI, wound infection, gastrointestinal complications, and re-exploration for bleeding.^10^ In addition, the obtained different results could be in consequence of the different cut points for the definition of prolonged LOS and ICU stay so that the predictive power of this risk score was higher for the prediction of longer ICU stay.^11^ Moreover, we have discussed before that sometimes patients’ longer stay in the ICU and/or hospital may be due to other reasons such as the surgeon’s routines and preferences.^21^

Differences in demography and medical history between European and Iranian cardiac patients may be the other important reason for the unacceptable performance of the EuroSCORE among Iranian CABG candidates. Especially noteworthy are lower age and higher frequency of unstable angina and recent MI in Iranian patients (Table 1). Adding other important risk factors and risk markers based on regional needs and patient characteristics in every country may enable the model to accurately predict outcome.^22^^, ^^23^

We used the additive EuroSCORE in this study because not only is its calculation easy but also its application for clinical purposes is feasible compared to the logistic model. Furthermore, the additive EuroSCORE is as reliable as the logistic EuroSCORE in predicting early and late outcome after CABG.^7^ Karthik et al.^24^ showed that the discriminative power was similar in both systems as measured by the c statistic. Additionally, most of our patients were in the low-risk group, and it appears from the literature that the additive EuroSCORE is accurate in low-risk cases and also in patients undergoing isolated CABG.^5^^, ^^24^^, ^^25^ Even some multi-center studies have demonstrated that in some circumstances, the additive EuroSCORE is superior to the logistic model.^5^^, ^^26^

## Conclusion

In conclusion, the EuroSCORE had a low discriminative power in predicting early morbidity and prolonged LOS and ICU stay in our study group. This finding shows that we cannot use the additive EuroSCORE in the current format as the sole measure for risk estimation in Iranian CABG patients. However, further multi-center designed studies with more patients are warranted before suggesting the creation of a modified model for an accurate prediction of outcome. 

## Notes

***This paper should be cited as:*** Najafi M, Sheikhvatan M, Sheikhfathollahi M. Discriminative Power of EuroSCORE in Predicting Morbidity and Prolonged Hospital Stay in an Iranian Sample Population. J Teh Univ Heart Ctr 2014;9(1):15-19.
